# Choosing the Right Differentiation Medium to Develop Mucociliary Phenotype of Primary Nasal Epithelial Cells *In Vitro*

**DOI:** 10.1038/s41598-020-63922-8

**Published:** 2020-04-24

**Authors:** Anja E. Luengen, Caroline Kniebs, Eva Miriam Buhl, Christian G. Cornelissen, Thomas Schmitz-Rode, Stefan Jockenhoevel, Anja Lena Thiebes

**Affiliations:** 1 Department of Biohybrid and Medical Textiles (BioTex), AME - Institute of Applied Medical Engineering, Helmholtz Institute, RWTH Aachen University, Forckenbeckstraße 55, 52074 Aachen, Germany; 2 Aachen-Maastricht Institute for Biobased Materials, Faculty of Science and Engineering, Maastricht University, Brightlands Chemelot Campus, 6167 RD, Geleen, The Netherlands; 3 Institute of Pathology, Electron Microscopy Facility, RWTH Aachen University Hospital, Pauwelsstraße 30, 52074 Aachen, Germany; 4 Clinic for Pneumology and Internal Intensive Care Medicine (Medical Clinic V), RWTH Aachen University Hospital, Pauwelsstraße 30, 52074 Aachen, Germany

**Keywords:** Tissue engineering, Respiratory tract diseases

## Abstract

*In vitro* differentiation of airway epithelium is of interest for respiratory tissue engineering and studying airway diseases. Both applications benefit from the use of primary cells to maintain a mucociliated phenotype and thus physiological functionality. Complex differentiation procedures often lack standardization and reproducibility. To alleviate these shortfalls, we compared differentiation behavior of human nasal epithelial cells in four differentiation media. Cells were differentiated at the air-liquid interface (ALI) on collagen-coated inserts. Mucociliary differentiation status after five weeks was analyzed by electron microscopy, histology and immunohistochemistry. The amount of ciliation was estimated and growth factor concentrations were evaluated using ELISA. We found that retinoic-acid-supplemented mixture of DMEM and Airway Epithelial Cell Growth Medium gave most promising results to obtain ciliated and mucus producing nasal epithelium *in vitro*. We discovered the balance between retinoic acid (RA), vascular endothelial growth factor (VEGF), epidermal growth factor (EGF) and fibroblast growth factor β (FGF-β) to be relevant for differentiation. We could show that low VEGF, EGF and FGF-β concentrations in medium correspond to absent ciliation in specific donors. Therefore, our results may in future facilitate donor selection and non-invasive monitoring of ALI cultures and by this contribute to improved standardization of epithelial *in vitro* culture.

## Introduction

In the respiratory tract, a specialized epithelium covered with motile cilia plays a major role in cleansing of the airways and maintaining the lung’s immunoprotection. In a mechanism called mucociliary clearance, dust, pathogens and other foreign particles are bound in cell-produced mucus that is transported mouthwards by cilia movement. Several ways exist in which this important defense and self-cleaning mechanism can be impaired^1^. Smoking, chronic obstructive lung disease and lung cancer are very common causes^2^. In addition to damage caused by the carcinoma itself, bronchotracheal cancer or spreading carcinomas of adjacent origin often lead to inoperable airway stenoses requiring stent implantation as palliative treatment option^3^. This procedure merely represents treatment of symptoms to improve quality of life^4^. It usually is accompanied by loss of mucociliary clearance function throughout the stent luminal surface and an increased risk of pneumonia and treatment failure^3^. To overcome these limitations in treatment of patients suffering from irresectable airway stenosis, research focuses on development of suitable tracheal replacements^5^.

Regenerative medicine promises autologous and tailor-made tissue substitutes without the need of immunosuppression^5^. For this reason, more and more tissue engineered tracheal replacements have arisen in recent past^6,7^. Nevertheless, these strategies still face challenges regarding vascularization, mechanical stiffness and proper epithelization^8,9^. To date, an ideal tracheal replacement has not been developed yet^3,5^. Although an autologous tissue composite derived from the patient’s forearm was currently stated as the only long-term working solution due to its pre-vascularization, it still suffers from missing mucociliary clearance^5^. Restoration of the latter is particularly hindered by insufficient quantity and proliferation of harvested cells, their early senescence and the need for an autologous but healthy cell source^10^.

Tracheal or bronchial epithelial cells derived from brush biopsies have frequently been used^11^ although patients requiring tracheal replacement may often not exhibit healthy epithelium in lower respiratory tract due to their disease and smoking history^12^. Epithelial cells from nasal origin may serve as surrogate for tracheal epithelial cells, especially as they can be obtained in less invasive procedures^11,13^. Although freshly isolated primary airway cells and fully differentiated respiratory epithelial cell cultures providing limited donor variety are commercially available, these are very expensive and not affordable for many research institutes^13^. Therefore, *in vitro* culture of functional respiratory epithelium represents a key factor in current respiratory tissue engineering approaches^10^.

Independent of their relevance for tissue engineering, respiratory epithelial *in vitro* systems have increasingly gained importance in recent past as legitimate alternative to expensive and often hardly transferable animal models that additionally raise ethical concerns^14,15^. Investigation of respiratory diseases and influences of various environmental factors on the airways has reached new levels via the possibility of using *in vitro*-differentiated airway epithelia^15,16^. These model platforms make use of a broad spectrum of cell sources and cultivation protocols^16^. Immortalized cell lines or tumor cells promise to overcome limitations of primary cell cultures regarding reproducibility and standardization, but they suffer from incomplete differentiation and lack of mucociliary phenotype^17^. Papazian *et al*. stated in 2016 that although *in vitro* differentiation of primary human respiratory epithelial cells (HREs) is time-consuming and complex, they “may be the best choice for studies of airway epithelium”^17^. Standardization and validation of methods are necessary to ensure that animal testing can progressively be replaced^14,16^. Zscheppang *et al*. criticized the use of heterogeneous protocols, cells, scaffolds and media for pulmonary *in vitro* models by different research groups and the resulting complications in inter-laboratory reproducibility^16^. Focusing on the cell culture media issue, it is consensus to use different media compositions for proliferation and differentiation of respiratory epithelial cells^18^. For both applications, a broad variety of commercially available or less expensive lab-specific media exists^19,20^. Some of the best-known manufacturers include Epithelix, Lifeline Cell Technology, Lonza, PromoCell and STEMCELL Technologies. Detailed media composition is confidential in most cases, making search for causes of discrepancies between results of different laboratories rather difficult.

For these reasons, we decided to evaluate differentiation behavior of primary human nasal epithelial cells in various differentiation media and analyze medium constituents. The media investigated included a modified version (mAir) of Airway Epithelial Cell Growth Medium (AECGM, PromoCell), PneumaCult-ALI Medium (Pneu, STEMCELL Technologies), MucilAir Culture Medium (Epi, Epithelix) and a mixture of Endothelial Cell Growth Medium 2 (EGM2, Promocell) with MucilAir Culture Medium (EMM). PromoCell provides detailed information on AECGM and EGM2 composition, while for PneumaCult-ALI medium supplements are only partially known. MucilAir ingredients are not disclosed. To evaluate differential cell behavior in these media designed for Air-liquid interface (ALI) culture, cells were seeded on collagen-coated inserts and proliferated under submerged conditions. ALI conditions to allow mucociliary differentiation were established after one week. After four weeks, epithelial differentiation status was analyzed by electron microscopy, histology and immunohistochemistry. Media composition and media-dependent cell signaling with respect to retinoic acid (RA), vascular endothelial growth factor (VEGF), epidermal growth factor (EGF), platelet derived growth factor BB (PDGF-BB), β nerve growth factor (β-NGF), stem cell factor (SCF), tumor necrosis factor α (TNF-α), fibroblast growth factor β (FGF-β) and transforming growth factor β (TGF-β) was investigated via enzyme-linked immunosorbent assay (ELISA).

## Results

Several specialized media for ALI culture of respiratory epithelium are available on the market promising reproducible mucociliary differentiation of primary cells *in vitro*. Nevertheless, we observed differences in epithelial phenotype after 4 weeks of ALI culture when using mAir, Pneu, Epi or EMM as differentiation medium. Additionally we found these differences to be highly donor-dependent. In summary, ALI culture in mAir led most reproducibly to mucociliary differentiation while in Pneu, Epi or EMM, HREs more often showed an undifferentiated phenotype.

### Medium influence on ciliation

**Figure 1:**
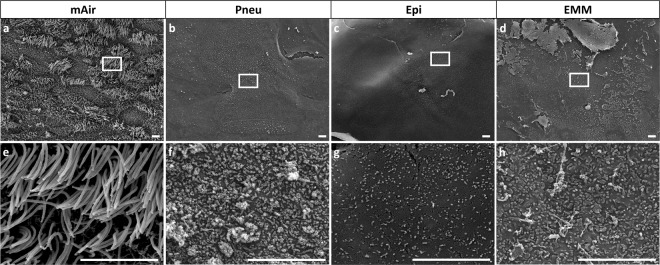
Ciliation after four weeks of ALI in different media (SEM). (**a**–**h**) SEM revealed cilia formation using mAir and microvilli formation without ciliation in Pneu-, Epi- and EMM-cultures. Representative images of two different magnifications are shown. Scale bar: 5 µm.

**Figure 2:**
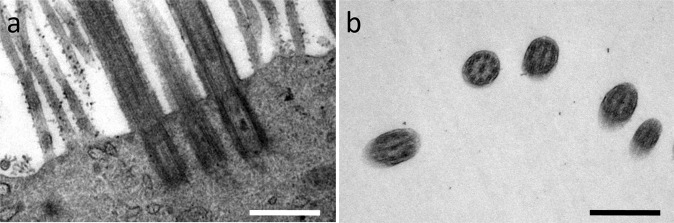
Cilia analysis of mAir-cultures (TEM). (**a**) Detection of basal bodies confirmed cilia formation; (**b**) Identification of central microtubuli doublets verified presence of secondary cilia. Representative images are shown. Scale bar: 500 nm.

Cilia formation was analyzed by SEM (Fig. 1). Using mAir led to cilia formation in HRE cultures (Fig. 1(a,e)) for most donors. We could detect characteristic 9 + 2-configuration of microtubuli and presence of basal bodies by TEM, which verified presence of motile cilia (Fig. 2). In Pneu-, Epi- or EMM-cultures, cell layers with microvilli lacking ciliation were most abundant (Fig. 1(b–d,f–h)). Figures show representative images. Cilia formation failed in mAir-cultures for two of seven donors while for Pneu and Epi, cilia could be observed with SEM in one of seven donors each. One Pneu-sample was lost due to contamination. For EMM, two of five donors developed cilia. SEM images and PAS reaction of all samples as well as immunohistochemical analysis of the untypical outcomes are shown in supplementary materials (see supplementary material S1 – S3).

**Figure 3:**
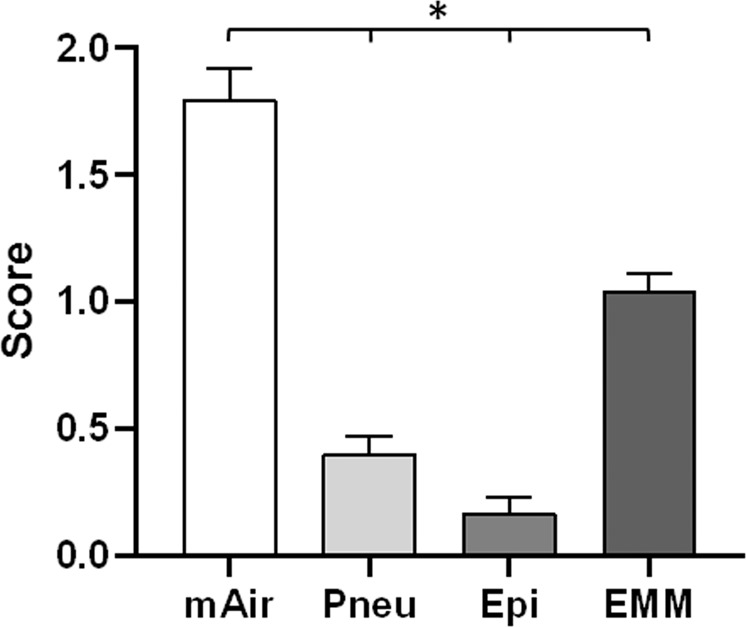
Ciliation scoring in ALI-cultures using different media. Scoring results showed statistically significant differences between all media. Results are expressed as mean ± SD following Gaussian error propagation. Statistically significant differences (p < 0.05) are indicated by *.

Estimation of cilia amount in SEM images by independent observers revealed significantly increased ciliation for mAir (mean score 1.80 ± 0.12, Fig. 3). Additionally, ciliation in EMM was significantly increased (mean score 1.04 ± 0.07) compared to rather low cilia formation in Pneu (mean score 0.40 ± 0.07) and Epi (mean score 0.17 ± 0.06).

### Medium influence on mucus production and expression of epithelial cell markers

**Figure 4:**
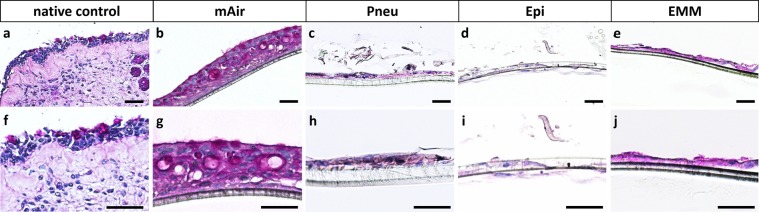
PAS reaction of mucociliary differentiation. (**a,f**) PAS reaction confirmed presence of glycogens, mucopolysaccharides and glycoproteins in human nasal concha tissue; (**b**–**e,g**–**j**) PAS reaction indicated cell multilayers and confirmed mucus production in mAir-cultures while using other media led to cell monolayers lacking mucociliary differentiation. Representative pictures of two different magnifications are shown. Scale bar: 40 µm.

To detect goblet cells and mucus production, PAS reaction was performed on cross sections of ALI-cultures and human nasal concha tissue as positive control (Fig. 4). For mAir-cultures, characteristic pseudostratified arrangement of respiratory epithelium with mucus secreting goblet cells and basal cells could be observed in most cases (Fig. 4(b,g)). Pneu-, Epi- or EMM-cultures revealed cell monolayers without mucus production or typical epithelial arrangement (Fig. 4(c–e,h–j)).

**Figure 5:**
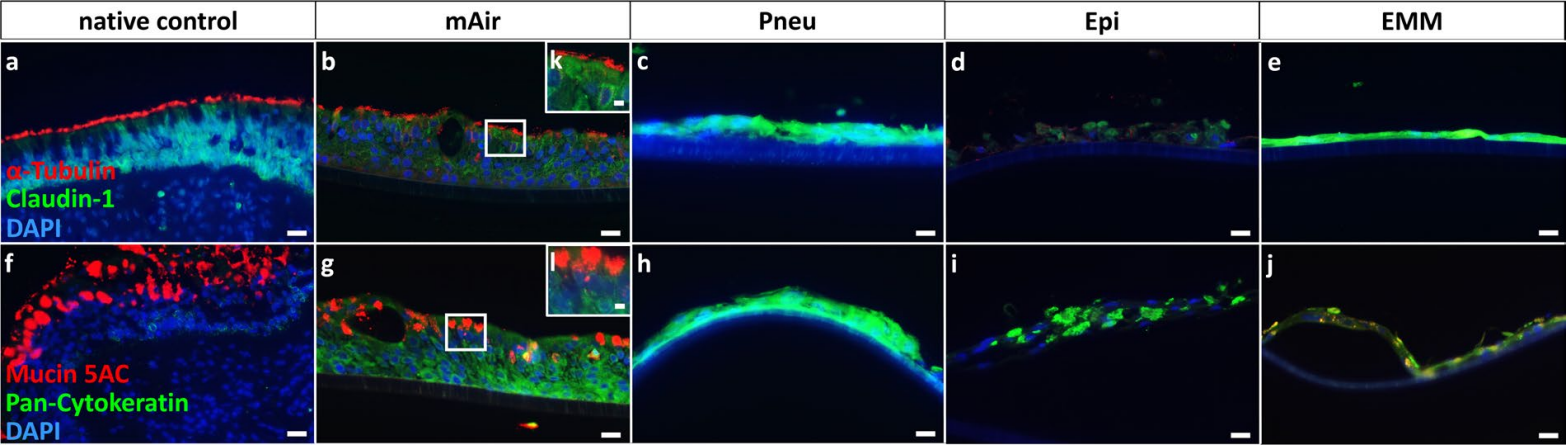
Immunohistochemical staining for differentiation status after four weeks of ALI in different media. (**a**–**e,k**) Claudin-1 (green) indicated tight junction formation in human nasal concha tissue and ALI-cultures. α-tubulin (red) visualized cilia in native tissue and mAir-cultures; (**f**–**j**,**l**) pan-cytokeratin (green) detected a wide range of keratins in all cultures and Mucin5AC (red) showed presence of goblet cells and mucus in the native control and in cell layers cultured in mAir. DAPI (blue) stained cell nuclei. Representative pictures are shown. Scale bar: 20 µm.

Immunohistochemical staining of typical epithelial cell markers was performed to confirm epithelial differentiation status. The staining included α-tubulin as one of two microtubuli monomers for staining of cilia, claudin-1 as major tight junction component to monitor barrier formation, mucin5AC as mucilaginous protein for mucus detection and pan-cytokeratin as marker for epithelial cytoskeleton^21^. In mAir cultures, staining for all four epithelial cell markers was positive and comparable to native human nasal tissue (Fig. 5(a,b,f,g,k,l)). Cilia formation, presence of goblet and basal cells, mucus secretion as well as tight junction formation could be verified. Immunohistochemial stainings of all mAir cultures are shown in supplementary materials (see supplementary material S4). Cell monolayers found in Pneu-, Epi- and EMM-cultures were negative for α-tubulin- and mucin5AC-staining but showed strong signals for claudin-1 and pan-cytokeratin, although cell arrangement and appearance of those signals differed from stainings known for epithelial tissue (Fig. 5(c–e,h–j)).

### Molecular analysis of growth factors and RA

**Figure 6:**
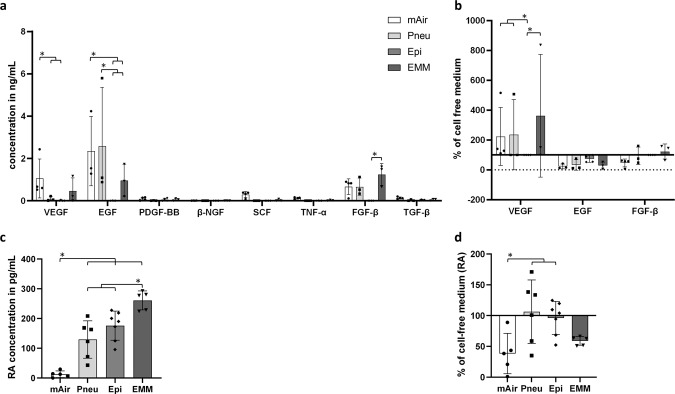
ELISA for human growth factor and RA analysis. (**a**) Concentrations of growth factors VEGF, EGF, PDGF-BB, β-NGF, SCF, TNF-α, FGF-β and TGF-β in different media in ALI after four weeks (selected donors); (**b**) VEGF, EGF and FGF-β concentrations from (**a**) normalized to cell-free medium (selected donors); (**c**) RA concentrations in different media in ALI after four weeks (all donors); (**d**) RA concentrations normalized to cell-free medium (all donors); The absorbance was measured at a wavelength of 450 nm. Error bars show standard deviation. Statistical significance indicated by * shows significance (p < 0.05) between mean values.

Media were analyzed by ELISA regarding growth factor and RA content to study influences of differentiation media on cell signaling (Fig. 6). RA content was analyzed for all HRE-cultures. As different donors showed different behaviors regarding cilia development, we decided to consider only those donors for growth factor analysis that showed ciliation in at least one medium. By this selection we wanted to eliminate donor-dependent impacts on ciliation from ELISA analysis. Hence, we were able to receive more meaningful results regarding the impact of media and their supplements on ciliation. Therefore, only donors with a ciliation score above 1 in at least one medium were chosen for growth factor analysis (supplementary materials S5).

ELISA revealed highly differing levels of growth factors for the culture media used. Significantly higher concentration of VEGF was found in mAir (1.06 ± 0.8 ng/mL) compared to Pneu (0.09 ± 0.09 ng/mL) and Epi (0.01 ± 0.02 ng/mL). Significantly lower concentration of EGF was observed in Epi (0.00 ± 0.00 ng/mL) and EMM (0.96 ± 0.62 ng/mL) compared to mAir (2.35 ± 1.34 ng/mL) and Pneu (2.59 ± 2.27 ng/mL). FGF-β concentration in EMM (1.24 ± 0.42 ng/mL) was significantly higher in relation to Epi (0.00 ± 0.00 ng/mL) (Fig. 6(a)). In relation to medium control incubated for one day in absence of cells, VEGF concentration in mAir (224 ± 169%), Pneu (202 ± 219%) and EMM (329 ± 364%) significantly increased in comparison with Epi (25 ± 42%) (Fig. 6(b)). Growth factors PDGF-BB, β-NGF, SCF, TNF-α and TGF-β were close to the detection limit in either medium. Therefore, relations between concentrations in used media and cell-free media would be caused by mathematical calculation of very low concentration values for these five growth factors and hence not meaningful. For this reason, relative values are only given for VEGF, EGF and FGF-β in Fig. 6(b).

RA concentration was significantly lower in mAir (12.87 ± 9.78 pg/mL) than in all other media (Pneu 129.62 ± 57.37 pg/mL, Epi 175.84 ± 45.32 pg/mL) whereas EMM showed the significantly highest concentration (260.66 ± 28.36 pg/mL) (Fig. 6(c)). In addition, we observed a significant decrease of RA in mAir (38 ± 29%) and a non-significant decrease in EMM (59 ± 6%) compared to the other media (Pneu 106 ± 47%, Epi 96 ± 25%) (Fig. 6(d)).

**Figure 7:**
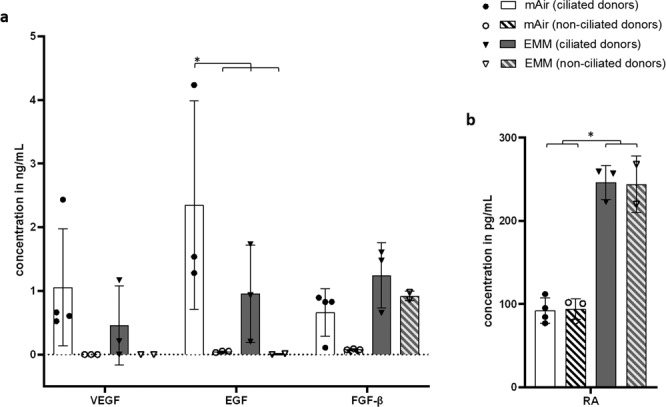
Comparison of growth factor and RA concentrations between ciliated and non-ciliated donors. (**a**) Growth factor concentrations for VEGF, EGF and FGF-β in mAir and EMM; (**b**) RA concentrations in mAir and EMM; Unfilled symbols and dashed bars represent donors without ciliation in any medium. Error bars show standard deviation. Statistical significance indicated by * shows significance (p < 0.05) between mean values.

To analyze the donor-dependent impact on growth factor content, we compared growth factor concentrations of donors with and without ciliation. As only VEGF, EGF, FGF-β and RA showed differences in previous ELISA analysis, we excluded other growth factors from this comparison. In addition, media EMM and mAir were selected in order to compare multiple ciliated and non-ciliated donors for each medium. In Fig. 7, concentrations of RA and growth factors VEGF, EGF and FGF-β are visualized for all donors for both media showing the highest degree of ciliation, mAir and EMM. Absence of cilia seemed to correlate with low concentration of VEGF and EGF (Fig. 7(a)). Inconsistent results can be found for FGF-β as its concentration for non-ciliated donors is lower than in ciliated donors for mAir but not for EMM. For RA, we could not detect differences between ciliated and non-ciliated donors but significant differences in RA concentration between both media (Fig. 7(b)).

## Discussion

Reproducibility and standardization are major challenges in the culture of functional respiratory epithelial *in vitro* systems used as disease models or for tissue engineering approaches. While ALI culture has been known to be essential for mucociliary *in vitro* differentiation of respiratory epithelium for years^22^, researchers have so far seeked to meet the epithelial need for an optimal medium composition by a broad variety of specialized differentiation medium protocols. On one hand, various commercially available differentiation media exist (for example provided by companies like Epithelix, STEMCELL Technologies, Lifeline Cell Technology, Lonza), while on the other hand, some laboratories use media based on their own recipes developed by experience^19,20^. Airway epithelial cells are known as very sensitive cell type regarding fast dedifferentiation *in vitro*, high senescence and low proliferation^23,24^. High seeding densities of more than $1.5\bullet {10}^{5}$ cells/cm^2^ are recommended in literature^18^. In general, differentiation media composition refers to a complex issue and requires a detailed view on cell type, species and application. Unfortunately, medium composition is not always publicly accessible for commercial products.

Here, medium influence on *in vitro* differentiation of HREs was examined with the media mAir (PromoCell), Pneu (STEMCELL Technologies), Epi (Epithelix) and EMM (PromoCell, Epithelix). The latter had previously been found to improve mucociliary differentiation and vascularization in tri-cultures of nasal epithelial cells with nasal fibroblasts and human umbilical vein endothelial cells in fibrin gels^21^.

In this study, we found mAir to be superior regarding mucociliary differentiation after four weeks of ALI. All donors showing ciliation developed cilia in mAir. In addition, ciliation frequency and amount were found to be highest in mAir. This was followed by EMM although differentiation frequency was significantly reduced. SEM results were supported by PAS reaction and immunohistochemial staining for epithelial cell markers. Most mAir cultures contained basal cells, ciliated cells, mucus producing goblet cells, tight junctions and epithelial cytoskeleton comparable to native nasal concha tissue.

The majority of HRE cultures in Pneu, Epi and EMM did not exhibit mucociliary differentiation but revealed formation of undifferentiated cell monolayers with atypical expression of claudin-1 and cytokeratin. Detection of the latter proves epithelial origin of the cell layers obtained after four weeks of ALI^25^ while claudin-1 expression gives evidence for tight junction development^26^. All samples underwent the same treatment regarding isolation, choice of proliferation medium, cell seeding and expansion in inserts. The media composition varied only from the point of switching to ALI conditions and cells derived from one donor were tested in all media. Consequently, basic requirements for epithelial differentiation were fulfilled for all samples when starting the ALI-culture. Nevertheless, mucociliary differentiation was not completed for most donors. Prolongation of ALI-culture could be an option to improve differentiation in these media. However, thickness of cell layers found for these media was rather small. This does not indicate successful differentiation after a longer ALI-period. On the contrary, the choice of proliferation medium may have a greater influence than the duration of ALI-culture. As the same proliferation medium was used for all cultures, it is possible that the combination of proliferation and differentiation medium impacts differentiation success. This is supported by the fact that we used AECGM as proliferation medium that is also part of mAir. Manufacturers STEMCELL Technologies and Epithelix provide an own proliferation medium to combine with Pneu and Epi. The choice of alternative proliferation media in the initial expansion phase in the inserts could also explain the successful use of Pneu and Epi in earlier studies reported in the literature^13,27,28^. Unfortunately, not all studies list the proliferation medium used. After all, it seems to be not strictly necessary to combine proliferation and differentiation media from the same company^13^. This would indicate the importance of optimized media setup for both phases in epithelial cell culture. Furthermore, the isolation technique used could have an influence on differentiation media needs and thus the differentiation success. Although enzyme-mediated digestion of surgical nasal tissue for up to 24 h at 4 °C is recommended in the literature as an initial step during isolation and has been proven to be successful^18,29,30^, nasal brushing techniques can be performed without this digestion and the associated incubation period^11,31^. We consider the impact of the enzymatic digestion step on the differentiation to be minor, as the cells from all donors proliferated well after isolation and had the same starting point when switching to ALI conditions and media variation. Moreover, most mAir cultures showed mucociliary differentiation following this treatment. However, while nasal concha tissue can be obtained from patients who are undergoing surgery anyway, nasal brushing techniques require volunteers and are therefore not available for all laboratories. The use of mAir can therefore enable a successful epithelial cell culture if nasal brushings are not available or tissue samples cannot be processed directly due to transportation issues. Further studies are necessary to evaluate impacts of manufacturer-dependent proliferation and differentiation media combinations and the connection between isolation techniques and media requirements.

Although differences in ciliation scoring between the four tested media were statistically significant, we still observed rather high donor variability regarding mucociliary differentiation. Cells derived from two of seven donors failed to develop cilia in any medium. Possible reasons might be an increased age beyond 50 years for one of the donors^32^ or the fact that the donor tissue was derived from patients with unknown medical history. Donor tissue was treated anonymously due to reasons of data protection. Although patients with severe respiratory diseases like asthma or cystic fibrosis were excluded, further aspects like drug treatments, allergic inflammations or nasal polyps could therefore not be taken into account^33^. It cannot be precluded that a patient’s lifestyle or medical history may have an influence on the isolated cells *in vitro*. Donor-dependent precondition of the nasal tissue has to be considered to explain these differences in demands on the differentiation medium. Primary cell differentiation, especially using respiratory epithelium, often suffers from variable outcomes with high donor-to-donor variability^34,35^. Donor-dependent variation was also observed in ELISA evaluation resulting in rather high standard deviations. To eliminate the donor-to-donor variability and to focus on media impacts on mucociliary differentiation, we decided to compare growth factor ELISA results only for cells derived from donors that showed ciliation in at least one medium with a threshold ciliation score of 1. As reasons for different ciliation behavior in other donors are unknown, it is not possible to evaluate media impact in these donors.

The mostly unknown composition of the two media Pneu and Epi complicates causal analysis of differential medium performance. Different components and growth factors have been described as beneficial for successful respiratory epithelium proliferation and differentiation like RA, EGF, insulin, transferrin, hydrocortisone, triiodothyronine and FGF-β. Supplementing the medium with bovine hypothalamus extract has been proven to be superior to serum-use^36^. Especially vitamin A-metabolite RA is regarded as a key component in development of several organs including the lung^37^. It also influences growth factor regulation and expression of extracellular matrix (ECM) proteins^38,39^.

According to the manufacturers’ information, mAir contains EGF, insulin, transferrin, hydrocortisone, epinephrine, RA and BPE. Hydrocortisone and Heparin are the only known supplements of Pneu while composition of Epi is not disclosed at all. EGM2 as part of EMM encloses FCS, EGF, FGF-β, insulin-like growth factor, VEGF, ascorbic acid, heparin and hydrocortisone.

Cozens *et al*. found an EGF concentration of 10 ng/mL most suitable for bovine bronchial epithelium^40^. As mAir was supplemented with EGF to a concentration of 10 ng/mL, our results support their findings. EGF concentration in EGM2 is specified by the manufacturer as 5 ng/mL resulting in a concentration of at least 2.5 ng/mL in EMM. We also found EGF in Pneu- but not in Epi-cultures. EGF concentration decrease in relation to cell-free medium suggests that HREs reduce the EGF level in medium by consumption. Observation of this effect in all other media supports this impression.

In mAir, we could detect little amounts of FGF-β that may originate from BPE^41^. FGF-β is known to be involved in epithelial repair and angiogenesis processes^42,43^. Therefore, combination of a low RA concentration with an EGF concentration of 10 ng/mL and FGF-β supplementation seemed to be most promising regarding differentiation success. FGF-β was also found in Pneu but seemed neither to be consumed or produced.

Literature reports a decreased differentiation capacity with RA concentrations higher than 100 nM^40^. This matches our results as mAir was the medium with the lowest RA content (50 nM). For Pneu and Epi, RA concentration was found to be an order of magnitude higher than in mAir with only little changes compared to the cell-free control. Nevertheless, highest RA concentration was detected in EMM resulting from supplementation with fetal calf serum^44^. As EMM showed better results in ciliation scoring than Pneu and Epi, we conclude that growth factor interactions might be able to balance high RA levels in media. Regulatory interactions between RA and VEGF, which plays an important role in vascularization, have been reported^38^. In correspondence with that, VEGF concentrations were highest in mAir and EMM. In earlier studies done by our group, synergies between epithelial differentiation and enhanced vascularization in a tri-culture model with nasal epithelial cells, nasal fibroblasts and human umbilical vein endothelial cells could be demonstrated^21^. In co-culture or pre-conditioning systems, supporting cell types can overcome medium limitations by providing necessary growth factors^45^. This may be an explanation for differences in outcome of differentiation media testing as EMM has been selected by Kreimendahl *et al*. to be the optimal medium for tri-culture studies^21^. Increase in VEGF concentration in mAir, Pneu and EMM with respect to the cell-free medium control indicates enhanced VEGF production. This may be a hint for the importance of vessel formation for epithelial differentiation process.

Growth factors PDGF-BB, β-NGF, SCF, TNF-α and TGF-β could not be measured at all or only at the detection limit. This suggests a minor role of these growth factors in mucociliary differentiation.

For RA and growth factors VEGF, EGF and FGF-β, we compared ELISA results between ciliated and non-ciliated donors in the most promising media mAir and EMM. VEGF and EGF concentrations were rather low in non-ciliated donors in comparison with samples that showed ciliation indicating an increased growth factor uptake. This could imply that non-ciliated donors were not able to maintain a certain growth factor level in the medium. Possible reasons are an overall decreased metabolism or a lower total cell count due to poor proliferation. The effect could also be observed for FGF-β in mAir but not in EMM. This difference might be caused by the higher initial FGF-β concentration in EMM. Since ciliation seemed to correspond highly to growth factor content in conditioned medium, media analysis may in the future offer a non-invasive way to monitor differentiation and possibly exclude donors at an early stage.

Further research has to be done to evaluate the ideal balance between growth factor concentrations, the temporal course of cytokine production in ALI-culture and the benefits in supplementing VEGF or FGF-β. In addition to that, future studies could focus on analysis of proliferation and differentiation media combinations to ensure increasing reliability and standardization of airway epithelial cell culture for tissue engineering applications and basic respiratory research.

In this study, we compared mucociliary differentiation in four separate differentiation media for primary nasal epithelial cells. We were able to reproducibly obtain pseudostratified ciliated epithelium including mucus producing goblet cells using a modified version of Airway Epithelial Cell Growth Medium. Nevertheless, our results were accompanied by high donor-to-donor variability often seen in primary cell culture. In summary, ELISA analysis of growth factor and RA content revealed the importance of balanced RA, VEGF, EGF and FGF-β concentrations in differentiation medium for respiratory epithelium. We could prove that growth factor content in medium corresponds to differentiation status. By this, we pointed out new ways to monitor primary ALI-cultures. In addition, our results may be helpful to classify primary cells regarding their differentiation capacity in the future.

## Materials & Methods

### Isolation and expansion

HREs were isolated from nasal conchae received from the Clinic for Otorhinolaryngology (RWTH Aachen University Hospital). The local ethics committee approved provision of nasal tissue (EK 067-18). Donors were between 10 and 40 years old with one exception being between 50 and 60 (see supplementary material S6).

Cell isolation was performed using a recently described protocol^21^. In short, the tissue was incubated in dispase (2.4 U/mL, Gibco) at 4 °C for 20–22 h. After dispase deactivation with Dulbecco’s Modified Eagle Medium (DMEM, Gibco) containing 10% fetal calf serum (FCS, Gibco) and 1% antibiotic/antimycotic (ABM, Gibco), the epithelial cells were scratched from the basal membrane using a cell scraper. Following filtration through a 100 µm cell strainer, the cell solution was centrifuged at 200 *g* for 5 min. Cells were expanded in T75 cell culture flasks in Airway Epithelial Cell Growth Medium (AECGM, PromoCell) with 0.1% Gentamicin (40 µg/mL, Rotexmedica) in a humidified incubator at 37 °C with 5% CO_2_. Exchange of culture medium was performed every 2–3 days.

### Cell seeding, proliferation and differentiation

All experiments were performed with cells in the first passage. Compared differentiation media are listed in Table 1. mAir consisted of a mixture of Airway Epithelial Cell Basal Medium (AECBM, PromoCell) and DMEM supplemented with bovine pituitary extract (BPE, 0.004 mL/mL), EGF (10 ng/mL), insulin (5 µg/mL), hydrocortisone (0.5 µg/mL), epinephrine (0.5 µg/mL), transferrin (10 µg/mL, all PromoCell), RA (50 nM, Sigma-Aldrich) and tranexamic acid (TA, 0.16 mg/mL, Carinopharm). For preparation of Pneu, PneumaCult-ALI Basal Medium (STEMCELL Technologies) was supplemented with PneumaCult-ALI 10x supplement, PneumaCult-ALI maintenance supplement, heparin (0.004 mg/mL), hydrocortisone (0.48 µg/mL, all STEMCELL Technologies) and TA (0.16 mg/mL, Carinopharm). MucilAir Culture Medium (Epithelix) was supplemented with TA (0.16 mg/mL, Carinopharm) to prepare Epi. EMM consisted of a mixture of MucilAir Culture Medium and EGM2 supplemented with TA (0.16 mg/mL, Carinopharm). For EGM2 preparation, Endothelial Cell Basal Medium 2 (PromoCell) was supplemented with FCS (0.02 mL/mL), EGF (5 ng/mL), FGF-β (10 ng/mL), insulin-like growth factor (20 ng/mL), VEGF (0.5 ng/mL), ascorbic acid (1 µg/mL), heparin (22.5 µg/mL) and hydrocortisone (0.2 µg/mL, all PromoCell). All media except predefined MucilAir Culture Medium were supplemented with 0.1% Gentamicin to avoid contamination. Cells derived from seven patients were subject of our investigation for all media except EMM (n = 5). They were harvested at 70% confluency with 0.05% Trypsin/0.02% EDTA in phosphate-buffered saline (PAN-Biotech) and seeded at a density of 150.000 cells/cm^2^ onto Transwell Inserts (PET membrane, 0.4 µm pore size, Corning) pre-coated with collagen IV from human placenta (100 µg/cm^2^, Sigma-Aldrich) in ThinCert Plates (Greiner Bio-One). Cells were cultured for proliferation under submerged conditions in AECGM, culture medium was changed every 2–3 days. Transepithelial electrical resistance (TEER) measurements were performed for cells derived from two donors to monitor barrier formation during the proliferation phase (see supplementary material S7). ALI conditions using the four differentiation media in parallel were established after one week. By this procedure, the cells from each donor were tested with all media.

**Table 1:** Differentiation media information. In the following, abbreviations are used for reasons of simplification.

Abbreviation	Medium	Medium composition	Manufacturer
mAir	modified AECGM	1:1-mixture of DMEM and AECBM with AECGM supplements without Triiodo-L-thyronine, Addition of 50 nM RA	Gibco, PromoCell, Sigma-Aldrich
Pneu	PneumaCult-ALI Medium	according to manufacturer’s instructions	STEMCELL Technologies
Epi	MucilAir Culture Medium	according to manufacturer’s instructions	Epithelix
EMM	EGM2-MucilAir-Mixture	1:1-mixture of MucilAir Culture Medium and Endothelial Cell Growth Medium (EGM2)	Epithelix, PromoCell

### Evaluation of epithelial cell differentiation

After four weeks of differentiation phase, cell differentiation status was analyzed by histologic and immunohistochemical methods. Samples and native human nasal concha tissue were fixed in 4% paraformaldehyde solution (PFA, Carl Roth) buffered with phosphate-buffered saline (PBS, Gibco). Following dehydration in an ascending ethanol series along with Roticlear (Carl Roth) and embedding in paraffin, 3 µm-thick sections were cut and stained by PAS reaction to analyze morphology of epithelial layers via bright field light microscopy. In short, dewaxed samples were hydrolyzed in 1% w/v periodic acid solution and stained with Schiff’s reagent (both Merck) after washing for 10 min each. Following washing in 35 °C warm tap water, cell nuclei were counterstained with Mayer’s hematoxylin (Sigma-Aldrich) for 5 min. Immunohistochemical stainings were also performed using PFA-fixed sections. Subsequent to dewaxing, sections were blocked with 5% normal goat serum (NGS, Dako) in 0.1% Triton-PBS for 1 h at room temperature. Then samples were incubated in primary antibody solutions overnight at 4 °C. Following three washing steps, sections were incubated in secondary antibody solutions for 1 h at 37 °C in the dark. Counterstaining of cell nuclei was achieved using DAPI (Molecular Probes) after further washing. All antibodies were diluted in PBS containing 1% w/v bovine serum albumin (BSA, Sigma-Aldrich) and 0.1% w/v sodium azide (Sigma-Aldrich). Primary antibodies included polyclonal rabbit anti-*pan*-cytokeratin (1:200, Acris), monoclonal mouse anti-mucin-5AC (1:800, Acris), polyclonal rabbit anti-claudin-1 (1:800, Biorbyt) and monoclonal mouse anti-acetylated tubulin (1:800, Sigma-Aldrich). For detection, fluorescently labelled secondary antibodies Alexa Fluor 488 goat anti-rabbit IgG and Alexa Fluor 594 goat anti-mouse IgG (both Invitrogen) were used.

An inverted fluorescence microscope (Axio Observer.Z1, Carl Zeiss) was used for observation.

Scanning (SEM) and transmission (TEM) electron microscopy was carried out by the facility for electron microscopy of the medical faculty of RWTH Aachen University. For SEM analysis, samples were fixed in 3% glutaraldehyde (Agar scientific), rinsed with 0.1 M sodium phosphate buffer (Merck) and dehydrated in an ascending ethanol series. Afterwards, samples were dried by critical point drying in liquid CO_2_ and were coated with a 10 nm gold/palladium layer (Sputter Coater EM SCD500, Leica). Microscopy was performed in a high vacuum environment at 10 kV acceleration voltage with an environmental scanning electron microscope (ESEM XL30 FEG, FEI). For TEM analysis, samples fixed in 3% glutaraldehyde were washed in 0.1 M Soerensen’s phosphate buffer (Merck) and post-fixed in 1% osmium tetraoxide (Carol Roth) in 17% sucrose buffer (Merck). Following dehydration in an ascending ethanol series, samples were incubated in a 1:1-mixture of Epon resin (Serva) and ethanol for 1 h before final incubation in pure Epon for 1 h. Samples were embedded in pure Epon at 90 °C for 2 h. Ultrathin sections (70-100 nm) were cut by ultramicrotome (Reichert Ultracut S, Leica) using a diamond knife (Diatome) and picked up on Cu/Rh grids (HR23 Maxtaform, Plano). To enhance contrast, sections were stained with 0.5% uranyl acetate and 1% lead citrate (both EMS). For observation, a Zeiss Leo 906 (Carl Zeiss) transmission electron microscope with an acceleration voltage of 60 kV was used.

### Molecular analysis

For evaluation of differential signaling patterns, an ELISA for eight human cytokines (Human Growth Factor ELISA Strip II, Signosis) including VEGF, EGF, PDGF-BB, β-NGF, SCF, TNF-α, FGF-β and TGF-β was performed on conditioned media at the end of ALI culture (week 4). An ELISA specific for RA (Retinoic Acid ELISA Kit, Cusabio) was used for analysis of RA content following manufacturer’s instructions. Results were evaluated according to manufacturers’ instructions and GraphPad Prism software (version 8.3.0) was used for statistical analysis. ELISA data for RA were analyzed by one-way analysis of variance (ANOVA) with Tukey’s post-hoc test while ELISA data for growth factors were analyzed by two-way ANOVA with Tukey’s post-hoc test. A value of p < 0.05 was considered statistically significant.

### Ciliation quantification

For comparison of amount of cilia developed when using different media, ten independent and well educated observers classified SEM images at two different magnifications (blinded for medium and donor) using the scoring system provided in Table 2. Mean values per image and afterwards for each medium were determined, standard deviation was calculated by Gaussian error propagation. GraphPad Prism software was used for one-way ANOVA with a p-value of p < 0.05 considered statistically significant.

**Table 2:** Scoring system for ciliation quantification.

Score	Ciliation
0	none
1	low
2	medium
3	strong
4	very strong

### Ethics approval and consent to participate

All methods described in this study were carried out in accordance with the relevant guidelines and regulations, including the Helsinki declaration and current guidelines in good laboratory and good scientific practice. The isolation and use of primary human cells was approved by the local ethics committee of the Medical Faculty of RWTH Aachen University, Germany (EK 067-18) after informed written consent. Informed consent was obtained from all patients and/or parents or legal representatives involved in the study prior to tissue donation.

## Supplementary information

Supplemantary materials.

## Data Availability

The datasets generated and analysed during the current study are available from the corresponding author on reasonable request.
