# Supplementary material for: Choosing the Right Differentiation Medium to Develop Mucociliary Phenotype of Primary Nasal Epithelial Cells In Vitro
Source: Sci Rep. 2020 Apr 24;10:6963. doi: 10.1038/s41598-020-63922-8 (PMC7181704; doi:10.1038/s41598-020-63922-8)
Supplement: Supplementary file 1 — Supplemantary materials. [file 41598_2020_63922_MOESM1_ESM.pdf]

Choosing the Right Differentiation Medium to Develop Mucociliary Phenotype of Primary Nasal Epithelial Cells In Vitro

*Anja E. Luengen[1,2], Caroline Kniebs[1,2], Eva Miriam Buhl[3], Christian G. Cornelissen[1,4], Thomas Schmitz-Rode[1], *Stefan Jockenhoevel[1,2], Anja Lena Thiebes[1,2],

[1] Department of Biohybrid and Medical Textiles (BioTex), AME - Institute of Applied Medical Engineering, Helmholtz Institute, RWTH Aachen University, Forckenbeckstraße 55, 52074 Aachen, Germany

[2] Aachen-Maastricht Institute for Biobased Materials, Faculty of Science and Engineering, Maastricht University, Brightlands Chemelot Campus, 6167 RD, Geleen, The Netherlands

[3] Institute of Pathology, Electron Microscopy Facility, RWTH Aachen University Hospital, Pauwelsstraße 30, 52074 Aachen, Germany

[4] Clinic for Pneumology and Internal Intensive Care Medicine (Medical Clinic V), RWTH Aachen University Hospital, Pauwelsstraße 30, 52074 Aachen, Germany

* Corresponding authors:

Univ.-Prof. Dr. med. Stefan Jockenhoevel
Department of Biohybrid & Medical Textiles (BioTex)
AME- Institute of Applied Medical Engineering
Helmholtz Institute
RWTH Aachen University
Forckenbeckstraße 55
52074 Aachen - Germany
E-Mail: jockenhoevel@ame.rwth-aachen.de
0049-241-8047478

Anja E. Luengen
Department of Biohybrid & Medical Textiles (BioTex)
AME- Institute of Applied Medical Engineering
Helmholtz Institute
RWTH Aachen University
Forckenbeckstraße 55
52074 Aachen - Germany
E-Mail: luengen@ame.rwth-aachen.de
0049-241-8047462

Supplementary Material

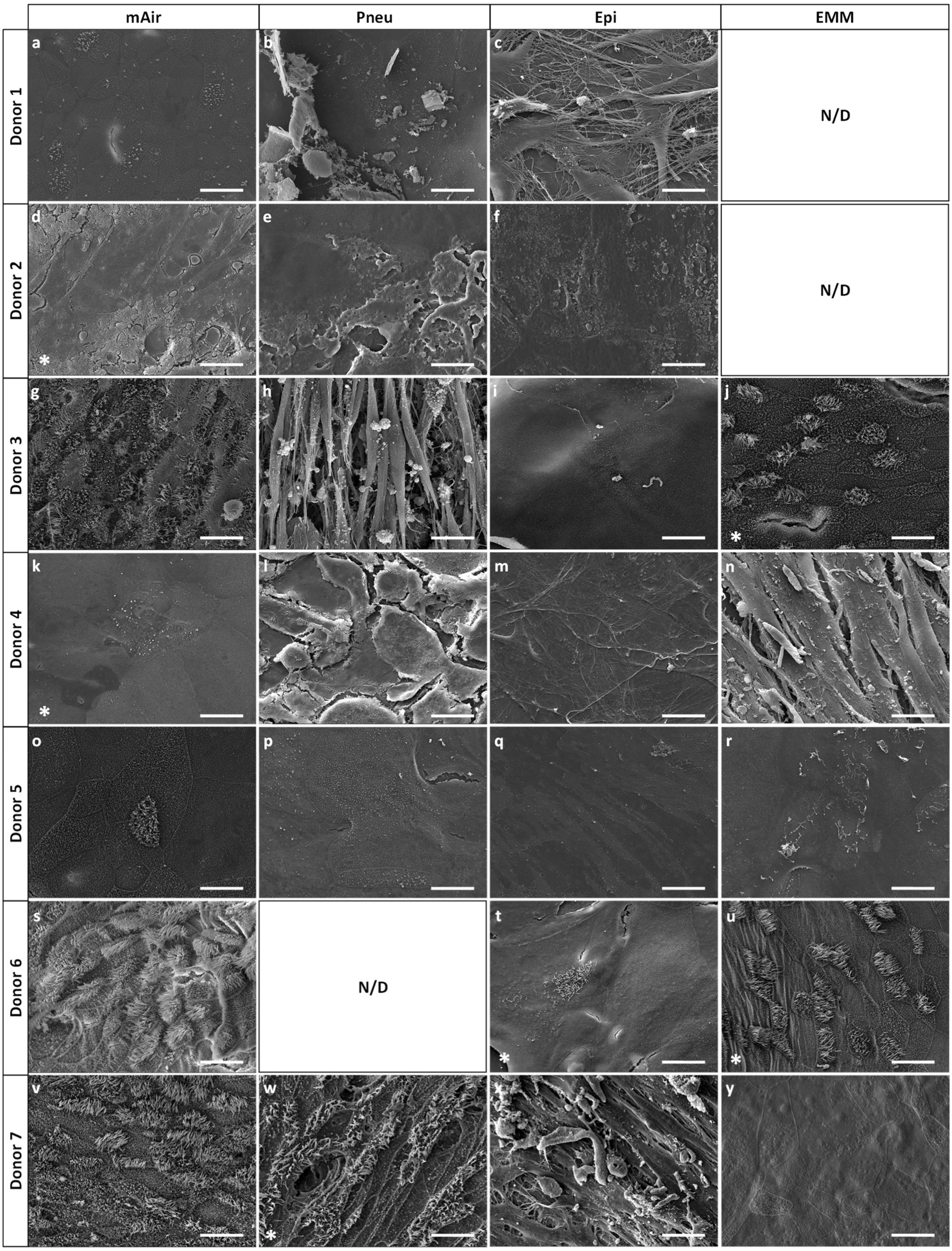

S1: SEM images of all donors for each medium. *Cells derived from seven patients were subject of our investigation for all media except EMM (n = 5). One Pneu-sample (donor 6) was lost due to contamination. (a, d, g, k, o, s, v): mAir-cultures; (b, e, h, l, p, w): Pneu-cultures; (c, f, l, m, q, t, x): Epi-cultures; (j, n, r, u, y): EMM-cultures; Untypical outcomes regarding the ciliation status are indicated by asterisks. (d, k): In two of seven cases, cilia formation failed in mAir-cultures; (w, t): Ciliation with Pneu or Epi was observed in one of seven cultures each; (j, u): Ciliated cell layers could be detected using EMM in two of five cases. Scale bar (a – y): 20 µm.*

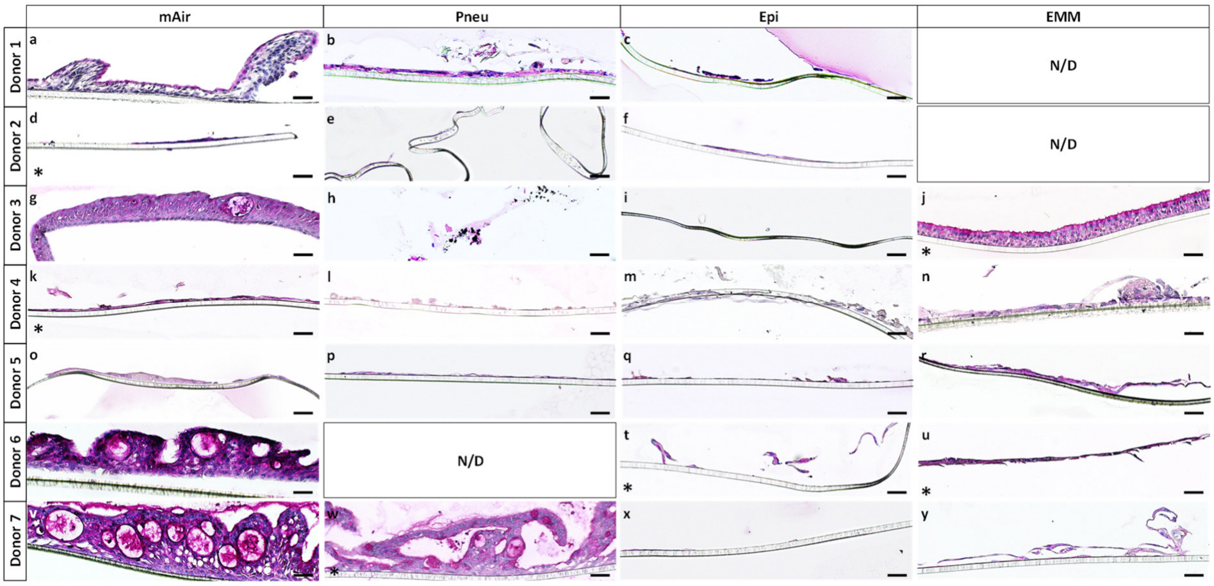

S2: PAS reaction images of all donors for each medium. *Cells derived from seven patients were subject of our investigation for all media except EMM (n = 5). One Pneu-sample (donor 6) was lost due to contamination. (a, d, g, k, o, s, v): mAir-cultures; (b, e, h, l, p, w): Pneu-cultures; (c, f, l, m, q, t, x): Epi-cultures; (j, n, r, u, y): EMM-cultures; Untypical outcomes regarding mucociliary differentiation are indicated by asterisks. (d, k): In two of seven cases, mucociliary differentiation could not be detected in mAir-cultures according to SEM results; (o): PAS reaction could not detect mucociliary differentiation although SEM analysis revealed cilia formation; (w): Mucociliary differentiation with cilia growing inside air channels could be proved in one of seven cultures using Pneu; (t): Untypical cilia formation in Epi observed in SEM analysis (see S1) could not be confirmed in PAS reaction; (j, u): Ciliated cell layers using EMM detected in SEM analysis in two of five cases could be confirmed in one case. Scale bar (a – y): 40 µm.*

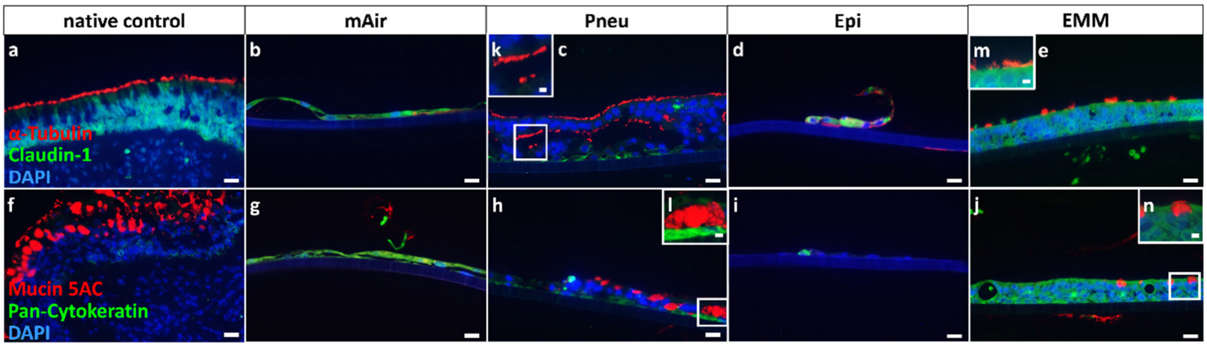

S3: Immunohistochemical staining of untypical cases for differentiation status evaluation. *(a, f): Claudin-1 (green) shows tight junction formation and α-tubulin (red) visualized cilia in native human nasal concha; (b): Lack of cilia formation was visible in two of seven cultures using mAir; (c, k): Ciliation in two directions and tight junction formation could be confirmed in one of seven Pneu-cultures; (d): α-tubulin staining could not confirm unusual cilia formation in Epi observed in SEM analysis (see S1); (e, m): Ciliated cell multilayers were detected in two of five EMM-cultures; (f): staining for pan-cytokeratin (green) and Mucin5AC (red) showed keratins, goblet cells and mucus in native tissue control; (g): Mucus production failed in two of seven mAir-cultures; (h, l): One of seven samples cultured in Pneu exhibited mucus production; (i): Untypical cilia formation in Epi observed in SEM analysis (see S1) was not accompanied by mucus production; (j, n): Two of five samples cultured in EMM featured mucus production. DAPI (blue) stained cell nuclei. Scale bar: 20 µm.*

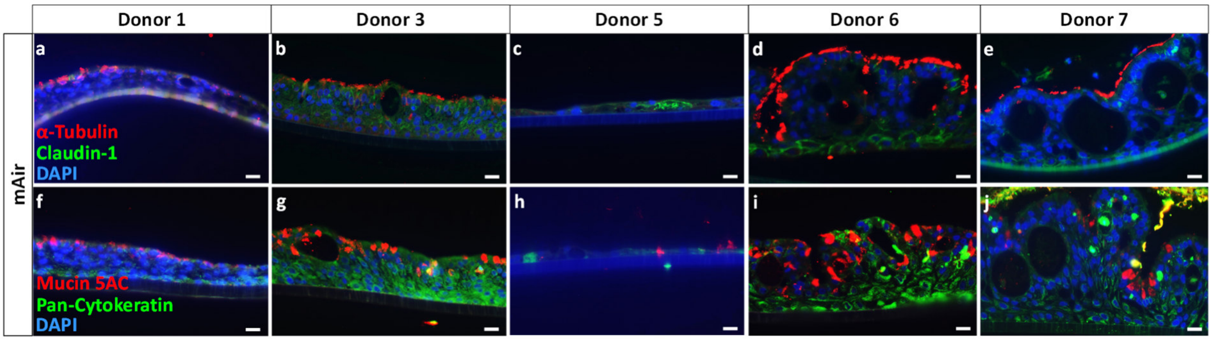

S4: Immunohistochemical staining of ciliated mAir samples for differentiation status evaluation. *(a – e): Claudin-1 (green) shows tight junction formation and α-tubulin (red) visualized cilia; (c): Although cilia could be found in SEM analysis, α-tubulin staining remained negative in mAir-sample of donor 5; (f – j): staining for pan-cytokeratin (green) and Mucin5AC (red) showed keratins, goblet cells and mucus; DAPI (blue) stained cell nuclei. Scale bar: 20 µm.*

S5: **Number of donors considered for growth factor ELISA**. *Four donors exhibited a minimum ciliation score above 1 in at least one medium.*

Medium	*Number of donors*
mAir	4
Pneu	3
Epi	4
EMM	3

S6: Age and ciliation outcomes of tissue donors. *For reasons of data protection and anonymization, age is given in a grouped presentation.*

**Donor**	**Age**	**Ciliation**			
		**mAir**	**Pneu**	**Epi**	**EMM**
**1**	30-40	Yes	No	No	-
**2**	50-60	No	No	No	-
**3**	20-30	Yes	No	No	Yes
**4**	20-30	No	No	No	No
**5**	10-20	Yes	No	No	No
**6**	20-30	Yes	contaminated	Yes	Yes
**7**	20-30	Yes	Yes	No	No

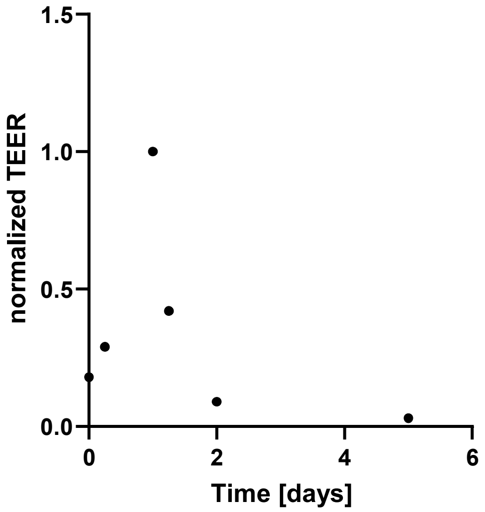

S7: Representative TEER measurement normalized to day 1. *Measurements were performed on cells in inserts during proliferation phase.*
